# Process Intensification by Coupling Gas Permeation and Membrane Contactor for Removing CO_2_ at Low Partial Pressure

**DOI:** 10.1021/acsomega.4c11526

**Published:** 2025-12-15

**Authors:** Felipe B. de S. Mendes, Cristina C. Pereira, Paulo C. Sedrez, Priscila Simões T. Amaral, Cristiano P. Borges

**Affiliations:** † Brazilian Navy Research Institute, Rio de Janeiro 21931-095, RJ, Brazil; ‡ COPPE/Chemical Engineering Program, 28125 Federal University of Rio de Janeiro, Rio de Janeiro 21931-095, Brazil

## Abstract

Effective solutions for removing CO_2_ are necessary to ensure the safety of crew members in confined spaces. Exploring enhanced process coupling strategies is a way to solve the important demand of maintaining low CO_2_ concentrations. Despite extensive inquiries into different approaches, a conclusive and ideal technology nonetheless remains unresolved. A novel method is presented that combines gas permeation with membrane contactors to effectively eliminate CO_2_ at low partial pressures. The mathematical models were validated using experimental data, and extensive computational evaluations of the coupled process were performed. The transport characteristics of gas permeation membranes demonstrate a prospective path for the development of high-performance membranes. More precisely, membranes with a gas permeance unit value of 1000 for CO_2_ and CO_2_/N_2_ selectivity of around 40 show a substantial decrease in the overall membrane surface area needed. Furthermore, the significance of process design in enhancing system performance is emphasized. The results suggest that using a single-stage flowsheet provides similar effectiveness as a two-step method while also simplifying operations. This study enhances the comprehension of CO_2_ elimination technologies and suggests a simplified, efficient resolution for real-world implementations.

## Graphical abstract

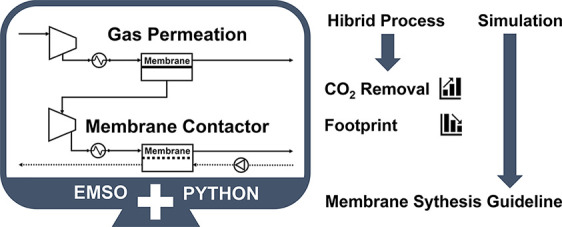

## Introduction

Confined spaces lack natural air circulation. The buildup of carbon dioxide (CO_2_) in these environments as a result of the exhalation of crew members is a significant issue. When CO_2_ exhaled by the crew is inhaled again, several symptoms can occur, such as mild confusion, blurred vision, loss of consciousness, or even death, at higher CO_2_ concentrations.^1^ Because forced ventilation of the atmosphere is not always possible, chemical processes are used to remove CO_2_, in order to maintain low CO_2_ concentration levels.^2,3^

Conventional regenerative methods for CO_2_ removal from closed spaces include column absorption using monoethanolamine (MEA) and molecular sieves using zeolites. However, these methods have limitations. Column absorption is susceptible to flooding and channeling,^4–6^ which emits noxious ammonia vapor. Molecular sieves require a large amount of space to remove the same amount of CO_2_ from a confined environment.^7,8^ Moreover, both processes require significant energy.^9^

Although conventional technologies exist for removing CO_2_ from confined spaces, researchers have investigated novel approaches to overcoming the limitations of these current methods. Membrane processes are compact and can easily be scaled up.^10^ Membrane contactors (MC) are viable alternatives to conventional regenerative processes owing to their high packing density, ease of operation, and scalability. A membrane contactor is an absorption process that generally uses porous membranes as the intermediate phase between the liquid and gas phases. The membrane maintains the phases apart and prevents mixing, whereas the liquid phase selectively absorbs the constituents of the gas phase.^11,12^ The solubility of gases in a liquid determines process selectivity.^10,13–15^ When using membranes, their interfacial area is known and constant, making it easier to control their performance compared to other processes, such as the packed column.

Several liquid absorbents have been used in membrane contactor processes, such as pure water, aqueous alkanolamines (monoethanolamine, diethanolamine and ethyldiethanolamine), and alkaline solutions.^16–22^ Mendes^17^ experimentally evaluated a membrane contactor with alkaline saline water to selectively separate CO_2_ from nitrogen. An inherent limitation of this method is the low partial pressure of CO_2_. However, implementing a preprocessing step to increase CO_2_ concentration addresses this issue.

The gas permeation process employs a dense membrane to selectively separate gases or vapors based on their interactions with the membrane.^10^ The parameter used to describe a dense membrane applied in gas permeation is its permeance, which is usually expressed in GPU[Fn fn1] (Gas Permeation Unit). Additionally, selectivity, which is typically calculated as the ratio of permeances, is another parameter used to indicate the difference in affinity between gaseous species and the membrane.

In this study, a gas permeation process before the membrane contactor is suggested to boost the driving force of the process. Hybrid processes are used to intensify CO_2_ removal because they potentiate the advantages of various processes.^23–26^ Consequently, the coupled process must be smaller than that of the standalone membrane contactor.

Using computer models, this study examines how gas permeation and a membrane contactor can work together to eliminate CO_2_ at low partial pressures. The study was performed computationally using models implemented in the software Environment for Modeling, Simulation, and Optimization (EMSO)^27^ simulation tools integrated with external Python codes. The main objective of this study was to determine the transport parameters (permeance and selectivity) of the gas permeation membrane that would achieve the smallest footprint while still removing a certain amount of CO_2_ from the atmosphere in small confined spaces.

The significance of this study lies in the development of innovative CO_2_ removal strategies for confined spaces, where conventional methods face limitations in space, energy use, and efficiency. By integrating gas permeation with membrane contactor technology, this hybrid approach enhances CO_2_ capture while reducing the footprint. Computational analysis provides insights into optimal transport parameters, guiding future experimental validation and real-world implementation, particularly in enclosed environments requiring effective air quality management

## Methodology

### Problem Description

Enclosed environments lack natural ventilation and, because of operational specifications, cannot have frequent forced ventilation to purify atmospheric air. Consequently, chemical processes are employed to eliminate CO_2_, which is emitted during human respiration cycles.

The hourly CO_2_ emission rate is constant and typical of the aforementioned application. A mathematical model that describes the process of removing CO_2_ was evaluated computationally in this study. The membrane contactor and coupling process of the membrane contactor and gas permeation were evaluated to remove CO_2_ at a fixed removal rate per hour.

### Model Description

The process units were modeled using a concentrated modeling approach. Figure 1 shows a schematic representation of the module units of the membrane contactor and the gas permeation. Subsequently, a detailed mathematical model for each process is explained.

**Figure 1:**
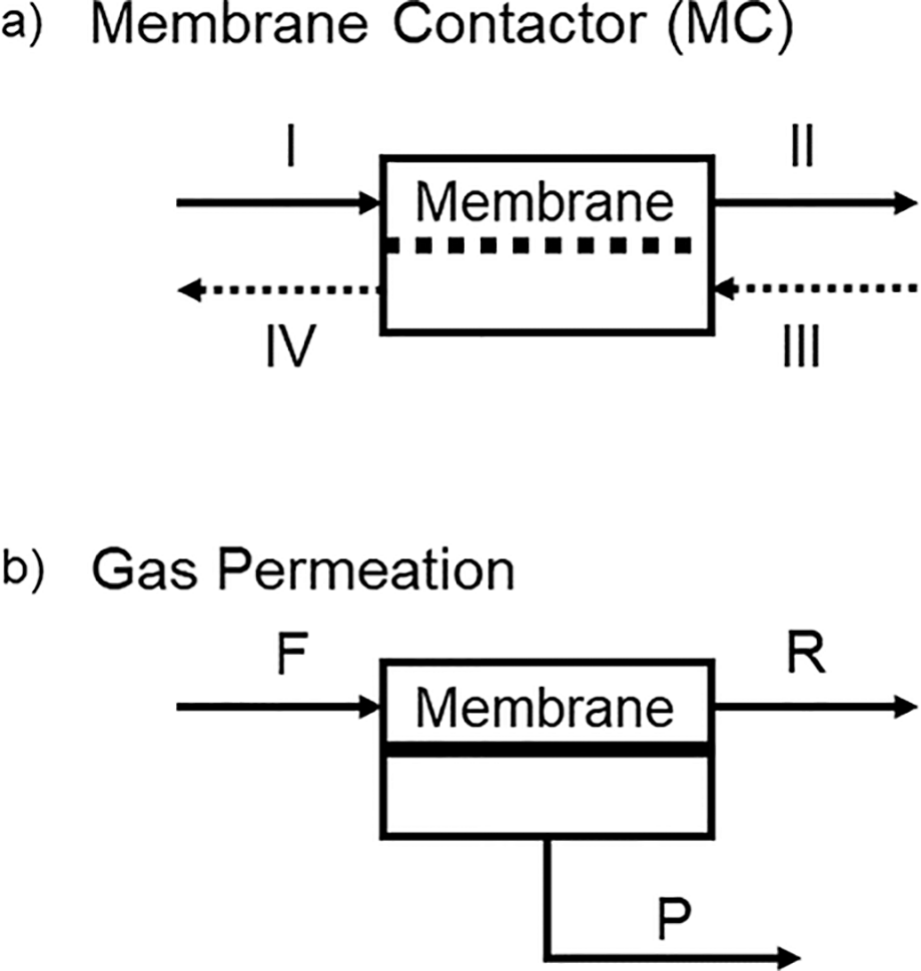
Schematic representation of the module units of the (a) membrane contactor and (b) gas permeation processes. Solid arrows correspond to gas streams and dashed arrows correspond to liquid streams.

#### Membrane Contactor

A concentrated mathematical model was utilized to represent the membrane contactors, which incorporated the mass balance by component in each phase. It was chosen to facilitate the process simulation, considering future integration with gas permeation. The following hypotheses were used:

- Liquid phase with constant temperature, pressure and flow rate.
- Gas phase as isothermal ideal gas.
- Component i transport based on the global mass transfer coefficient.
- Steady state.

The membrane contactor is shown schematically in Figure 1a. From this representation, it is possible to infer the mass balance equations for the liquid and gas phases, as outlined in eq 1a.

$$0=F_{I}.z_{I}\left(i\right)-F_{II}.z_{II}\left(i\right)-A_{CM}.KG\left(i\right).\Delta C_{m}\left(i\right)$$ 1a

$$0={(c}_{III}\left(i\right)-c_{IV}\left(i\right)).Q+A_{CM}.KG\left(i\right).\Delta C_{m}\left(i\right)$$ 1b

where eq 1a *a* is the molar balance in the gas phase and eq 1a *b* is the molar balance in the liquid phase. *F* is the molar flow rate of species *i*, *z* is the molar fraction of species *i*, *A*_*CM*_ is the area of the membrane, *KG* is the global mass transfer coefficient, Δ*C*_*m*_(*i*) is the logarithmic mean difference of the driving force of the process, *c* is the concentration of species, and *Q* is the volumetric flow rate of the liquid phase.

The mass transfer coefficient, which accounts only for the resistance in the liquid phase, was determined using the Yang and Cussler eq (1986), as documented by FUTSELAAR.^28^ The driving force of the process was determined by calculating the logarithmic mean difference between the concentrations of the gas and liquid currents using eq 2a. In this case, the logarithmic average considers the liquid and gas phases in countercurrent.

$$C0\left(i\right)=H\left(T_{Liq},S_{Liq}\right)\left(i\right).P_{I}.z_{I}\left(i\right)-{c_{Liq}}_{IV}\left(i\right)$$ 2a

$$CL\left(i\right)=H\left(T_{Liq},S_{Liq}\right)\left(i\right).P_{II}.z_{II}\left(i\right)-{c_{Liq}}_{III}\left(i\right)$$ 2b

$$\Delta C_{m}\left(i\right)=\frac{C0\left(i\right)-CL\left(i\right)}{\ln\left(\frac{C0\left(i\right)}{CL\left(i\right)}\right)}$$ 2c

The equations for determining the membrane area, overall mass transfer coefficient, and pressure drop in the gas phase are given in the Supporting Information. Furthermore, the model considered the geometric characteristics of the FiberFlo fiber module utilized by Mendes,^17^ as outlined in the Supporting Information.

#### Gas Permeation

Gas permeation was also modeled using a mass balance in the gas phase, which considered the following simplifying hypotheses:

- Gas phase as isothermal ideal gas.
- Feed pressure equal to retentate pressure.
- Steady state.

Figure 1b depicts the gas permeation module. Eq 3a represents the mass balance in the gas phase and eq 3b presents the permeate flow of component i across the membrane.

$$0=F_{A}.z_{A}\left(i\right)-F_{R}.z_{R}\left(i\right)-F_{P}.z_{P}\left(i\right)$$ 3a

$$0=J\left(i\right).A-F_{P}.z_{P}\left(i\right)$$ 3b

where *F* is the molar flow rate of species *i*, *z* is the molar fraction of species *i*, *A* is the gas permeation membrane area. The subscripts *F*, *R*, and *P* represent the Feed, Retentate and Permeate stream, respectively.

The permeation process only considers the solution-diffusion mechanism for the permeate gaseous species through the membrane. The Fick model was used to simplify the analysis, which is acceptable, even for ternary mixtures. The mathematical representation of the flow each component across the membrane is described by eq 4.

$$J\left(i\right)=\frac{Perm\left(i\right)}{l}.\left[P_{F}\left(i\right)-P_{P}\left(i\right)\right]$$ 4

where *J* is the flow rate of component *i* across the membrane, *Perm* is the permeability of component *i* in the membrane, *l* is the thickness of the membrane, *P* is the partial pressure of component *i*. The subscripts *F* and *P* are used to distinguish the feed and permeate streams, respectively.

### Model Implementation and Validation

Both models were implemented in the EMSO^27^ process simulator, which represents the Environment for Modeling, Simulation, and Optimization. The physical and chemical parameters of the gas phase were computed using the VRTherm thermodynamic program integrated with EMSO. The characteristics of the absorbent liquid were obtained using the model proposed by Mendes.^17^

#### Membrane Contactor

The mathematical model of the membrane contactor was validated using the experimental data previously presented by Mendes.^17^ The model proposed by the authors did not account for the chemical reaction occurring between CO_2_ and the absorbent liquid. Therefore, it is suggested that the impact of the chemical reaction be analyzed by incorporating it into an equation for the enrichment factor (*E*) while calculating the CO_2_ flux. Based on the existing knowledge, the enrichment factor is expected to be correlated with the temperature of the liquid. Therefore, it was assumed that the factor *E* is temperature-dependent and can be characterized by an Arrhenius equation with two parameters, *A* and *B*, as shown in eq 5.

$$E=A\cdot \exp\left(-\frac{B}{T}\right)$$ 5

eq 5 was fitted using the method of reducing the sum of squared differences between the experimental and calculated flux using the proposed model. EMSO software was integrated with Python scripts to use the optimization packages from the latter. The adjustment outcomes are shown in Figure 2, and the data are presented in the Supporting Information.

**Figure 2:**
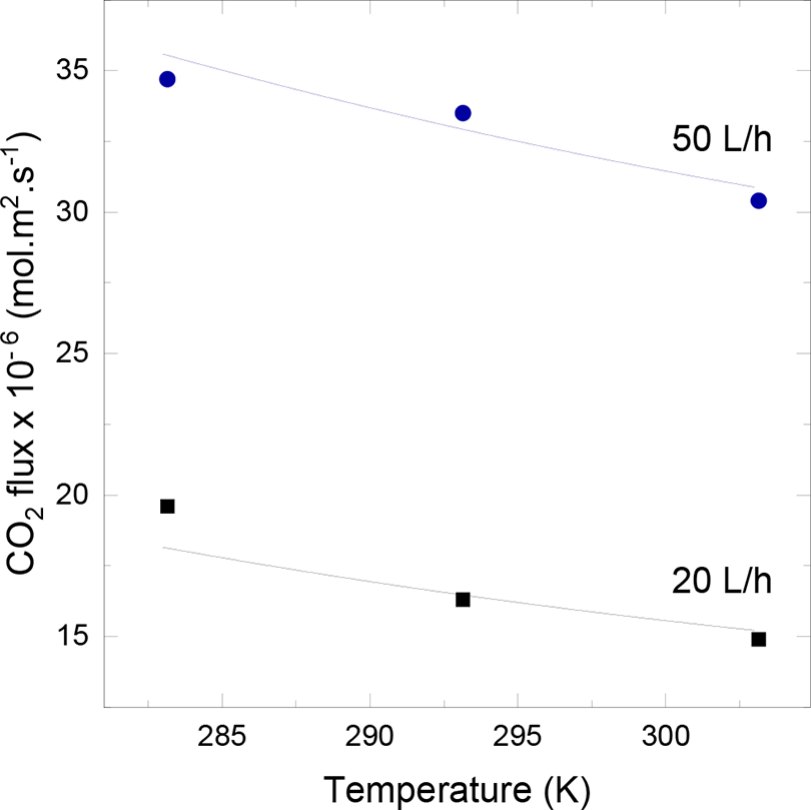
Membrane contactor model validation using temperature trend data at two different liquid flow rates.

Figure 2 demonstrates that the mathematical model effectively fits the experimental data and accurately replicates the decrease in CO_2_ flux as the temperature increases for both liquid flow rates used. The flux decreases as temperature rises because gas solubility in the liquid diminishes. The maximum absolute deviation was less than 10%, as shown in the Supporting Information.

#### Gas Permeation

The mathematical model for gas permeation was validated using the experimental data from Brinkmann et al.^29,30^ The authors collected data from experiments at different stage cuts and CO_2_ concentrations in the retentate and permeate streams of the flat-sheet membrane modules. Brinkmann et al.^29^ conducted experiments under different conditions using a flat-sheet membrane module to investigate the effect of permeation area on the concentration of CO_2_ in the retentate stream. They also examined the effect of the volumetric flow rate of the feed stream. The input parameters used to validate the mathematical model are presented in the Supporting Information.

The validation of the gas permeation model is shown in Figure 3. It is clear from Figure 3a and b that when the stage-cut values are below 10%, the model deviates less from the experimental data. Considering this threshold, the model exhibited a maximum error of 6%. Figure 3b displays the calculated stage-cut determined by the model. The region where the stage-cut ranged from 2 to 10% is marked by a dashed line.

**Figure 3:**
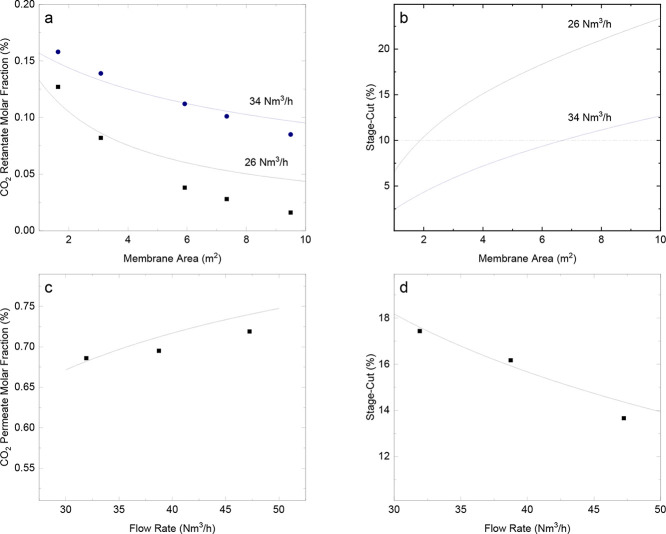
Validation of the gas permeation model: molar fraction of CO_2_ in the retentate vs membrane area for two different volumetric feed flow rates (a), stage-cut calculated vs membrane area for two different feed flow rates (b), molar fraction of CO_2_ in the permeate vs volumetric feed flow rate (c), and stage-cut vs volumetric feed flow rate (d). The solid lines represent the model predictions, while the gray region indicates the deviation between the model and previously reported data from Brinkmann et al.^29,30^ Adapted with permission from Brinkmann, T., & Pohlmann, J. (2012). Theoretical and experimental investigations of flat sheet membrane module types for high capacity gas separation applications. *Chemie Ingenieur Technik, 84*(8), 1237–1247, and Brinkmann, T., & Pohlmann, J. (2018). Characterization of a new flat sheet membrane module type for gas permeation. *Chemie Ingenieur Technik, 90*(11), 1667–1676. Copyright 2012 and 2018 Wiley-VCH Verlag GmbH & Co. KGaA.

Figure 3c shows that the proposed model exhibits a reduced deviation for lower CO_2_ concentrations in the permeate stream. The maximum error was 2.9%. Figure 3d presents a comparison between the experimental and simulated stage-cuts, with a maximum error of 5.2%.

In addition, according to the findings presented by Brinkmann et al.,^29^ who conducted simulations comparing modules with different geometries, it was observed that the gas permeation model proposed in this study conservatively estimates both the molar fraction of CO_2_ in the permeate and the stage-cut when compared to other models. The results of this comparison are presented in Supporting Information. The results demonstrate that the gas permeation model describes the behavior of the process by considering the molar percentage of CO_2_ at the permeate stream and stage-cut, particularly for values up to 10% of the latter. This is important because the gas permeation model is integrated into the membrane contactor model, and the coupling model also presents conservative estimations.

### Simulation

In all the simulations, the total power consumed by the processes was determined by summing the energy consumed by the chillers and flow equipment (pumps and compressors). The Reynolds number at the liquid stream of the membrane contactors was fixed at 60 to guarantee laminar flow inside the membrane module and observe the effects of other variables.

#### Single Processes

The membrane contactor module was simulated with a length of 1 m and the same packing density as that used experimentally by Mendes,^17^ using the configuration depicted in Figure 4a. The gas velocity per fiber was maintained at two different values, and different concentrations of CO_2_ were used in the feed stream. The total membrane contactor area was determined by replicating the modules in parallel, to ensure that the method achieved the desired fixed CO_2_ removal rate. The total liquid and gas flow rates of the processes were determined by multiplying the flow rate through one module with the total number of modules.

**Figure 4:**
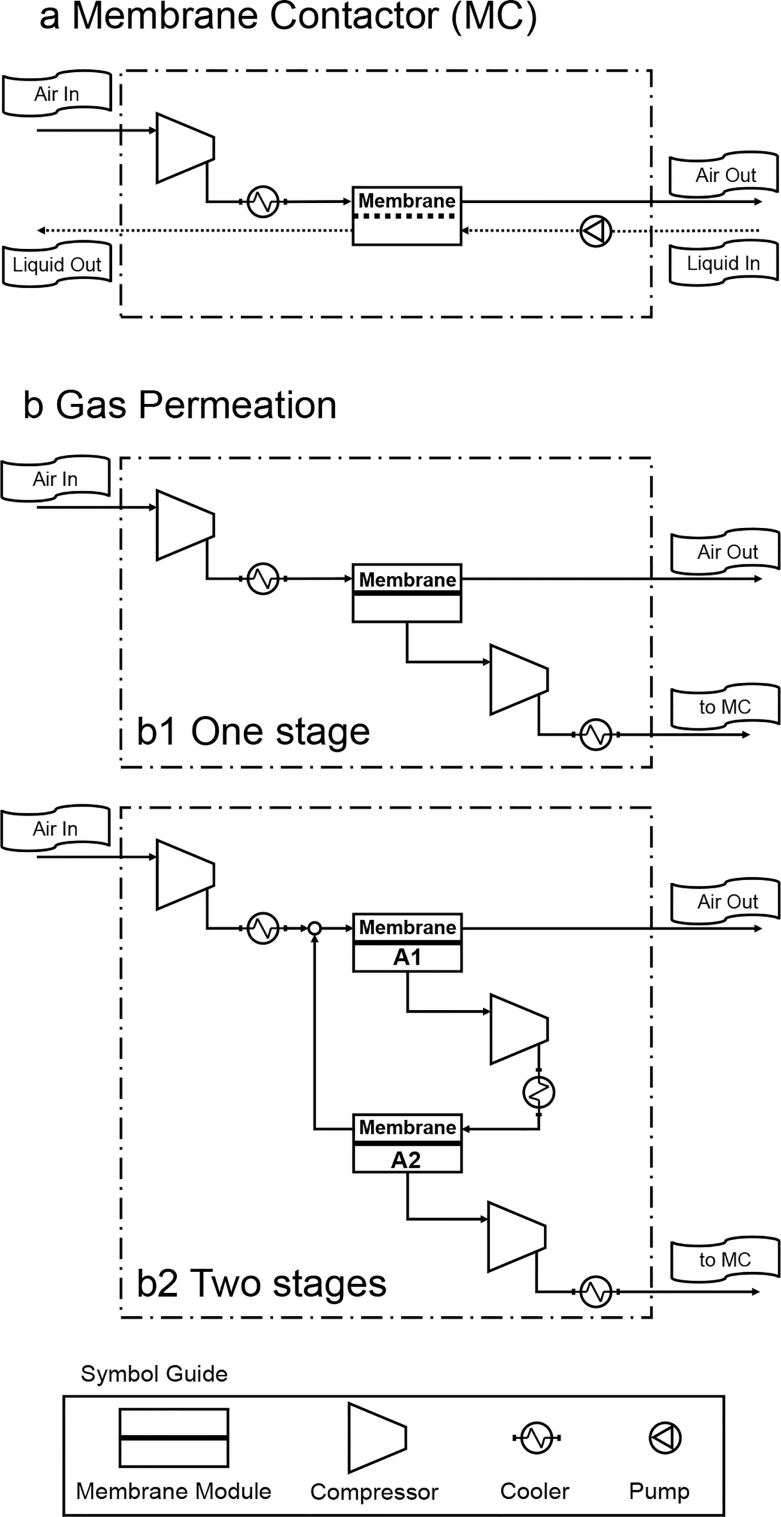
Flowhsheets of (a) membrane contactor and (b) gas permeation processes. Two gas permeation flowsheets are presented containing one stage (b1) and two stages where the membrane area ratio (A_1_/A_2_) is evaluated (b2).

The gas permeation process was simulated using two flowsheets, depicted in Figure 4b. The first flowsheet (Figure 4b1) contains one stage, while the other flowsheet (Figure 4b2) contains two stages, containing modulus with different areas computed as the area ratio A_1_/A_2_. The overall power consumption of the flowsheets is the sum of the power consumed by the compressors and the chillers.

Furthermore, six membranes were evaluated with permeability values of 100 or 1000 GPU and selectivity values of 10, 20, and 40 for CO_2_/N_2_, in accordance with commercially available membranes and existing literature.^31,32^ These membranes were labeled according to the nomenclature provided in Table 1. The O_2_/N_2_ selectivity remained constant at 2 for all cases.

**Table 1:** Gas Permeation Membranes Transport Properties Used at the Gas Permeation and Process Coupling Simulations^a^

nomenclature	permeance do CO_2_ (GPU)	selectivity CO_2_/N_2_
M-100/40	100	40
M-100/20	100	20
M-100/10	100	10
M-1000/40	1000	40
M-1000/20	1000	20
M-1000/10	1000	10

a Selectivity O_2_/N_2_ = 2 for all cases.

To evaluate gas permeation, the volume flow of the inlet stream before the compressor was kept constant at 500 m^3^/h at 298 K and 1 bar, and the gas permeation area was varied to obtain the desired stage cut for the process.

#### Process Integration

Process integration was performed using a Python script that initialized the EMSO models following the calculation approach depicted in Figure 5. Two methodologies were applied to evaluate the effect of the determined variable, that is, the feed flow rate at gas permeation or the gas velocity at the membrane contactor. It is important to note that the different methodologies are related only to the way the model is calculated and do not affect the final result.

**Figure 5:**
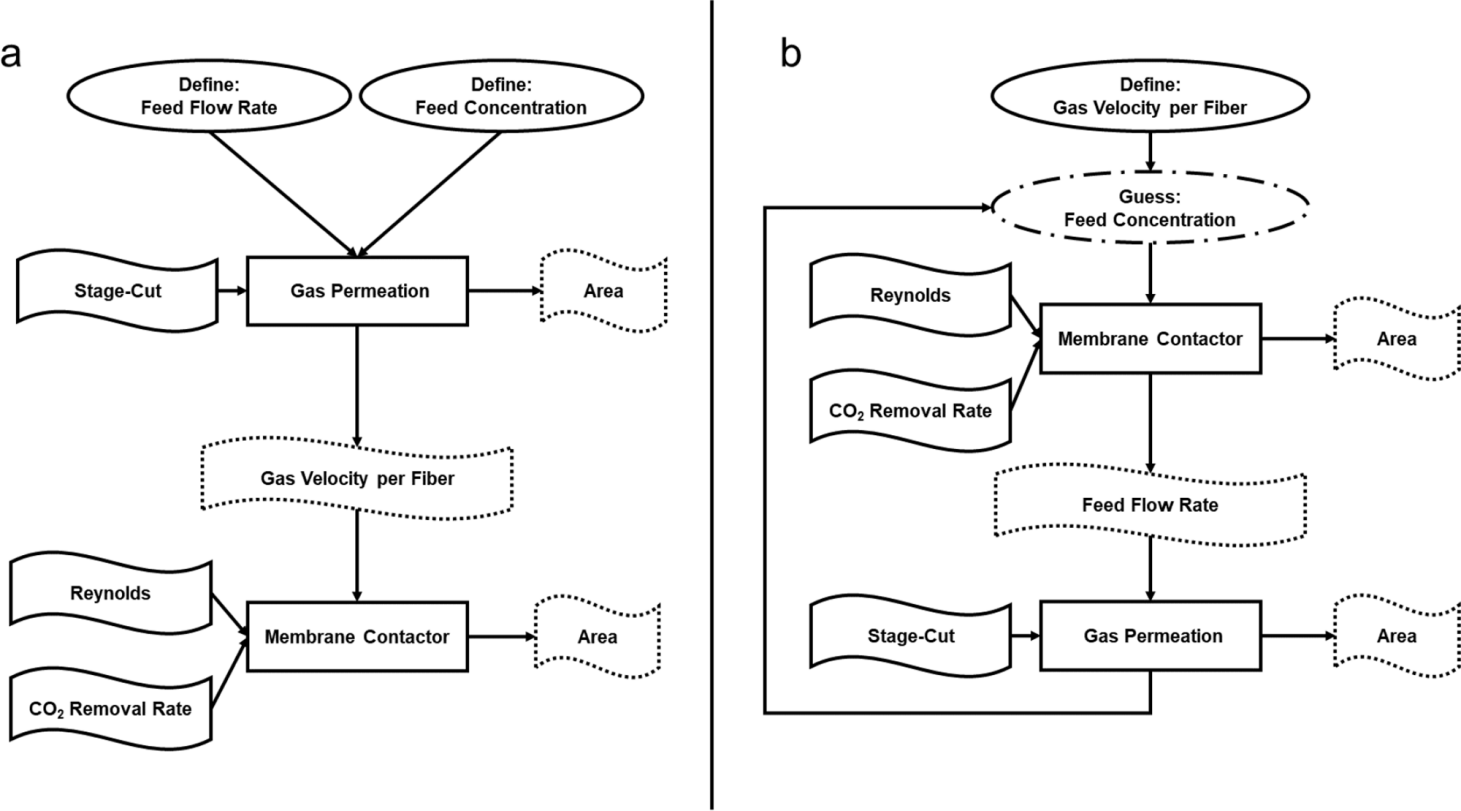
Two different methodologies were used to simulate the process coupling to evaluate the effect of the feed stream on the gas permeation of the gas velocity at the membrane contactor. Direct methodology (a) involves first calculating the gas permeation and then calculating the membrane contactor. Inverse methodology (b) involves first calculating the membrane contactor process and then the gas permeation process, which is repeated until convergence is achieved.

In the direct methodology (Figure 5a), the gas permeation process is determined prior to the membrane contactor in accordance with the physical sequence of process integration. The inlet flow rate for gas permeation was kept constant at 1000 m^3^/h at 298 K and 1 bar to simulate the high recirculation rate of the confined atmosphere. Consequently, the membrane area of the gas-permeation module was adjusted to achieve a predetermined stage-cut. The membrane contactors were simulated using the permeate stream from gas permeation.

In inverse methodology (Figure 5b), the membrane contactor was determined prior to gas permeation. The velocity of the gas per fiber in the membrane contactor inlet was maintained at a constant value. The membrane area of the membrane contactor was determined based on the initial estimation of the concentration of the gases being fed. The feed flow in this step was determined by calculating the number of contactors in parallel. Based on the predefined stage-cut, the total gas flow in the feed of the permeation process was calculated. Gas permeation was then calculated to determine the permeate stream concentration. Finally, the membrane contactor is recalculated. This process continued iteratively until the CO_2_ removal rate reached a tolerance of 10^–2^.

Process integration presupposes a reduction in the overall membrane area by combining the gas permeation process with the membrane contactor when compared with the latter alone. For the gas permeation process, two different flowsheets (Figure 4) and membrane transport properties (permeance and selectivity) were analyzed. For the membrane contactor, only the gas velocity in the feed stream was evaluated, and the other process variables were kept constant. It is important to mention that the gas inlet at the membrane contactor was maintained at 2 bar and 298 K.

## Results and Discussion

The membrane contactor and gas permeation processes were separately simulated to understand the influence of the main process variables. Subsequently, process coupling was evaluated by considering different gas-permeation membranes. The CO_2_ removal rate was maintained constant for all simulations, according to the requirements of the final application.

### Membrane Contactor: CO_2_ Feed Concentration

The effects of CO_2_ concentration on the membrane contactor process were evaluated using two gas velocities (0.03 and 0.31 m/s) per fiber inside the gas module, so the effect of the gas velocity could be evaluated. A 5-fold and 25-fold increase in the CO_2_ concentration resulted in an equivalent percentage decrease in the membrane area and power for both gas velocities. Increasing the concentration of CO_2_ to 5% resulted in an 80% reduction in the area and power. However, increasing the concentration to 25% led to a 96% drop.

Increasing the CO_2_ concentration in the inlet stream led to a reduction in the membrane area (Figure 6a), power (Figure 6b), and liquid and gas flow rates (Figure 6c,d). Based on the results presented in Figure 6, it is imperative to examine the integration of contactors with gas permeation. For this analysis, one should consider the total membrane area, that is, the gas permeation and membrane contactor area, and the overall power spent on the process.

**Figure 6:**
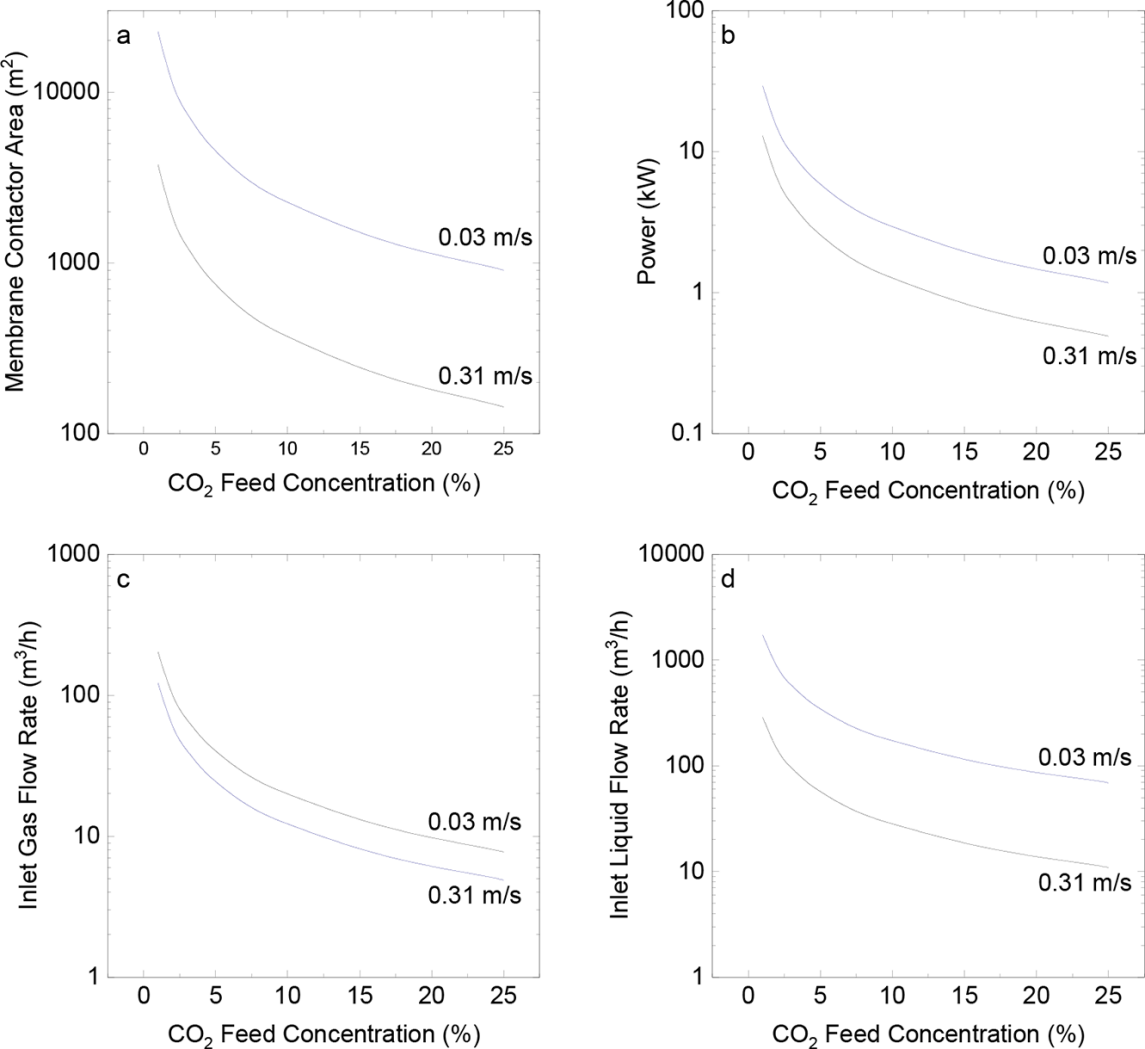
Impact of CO_2_ feed concentration on various factors: (a) membrane contactor area, (b) total power consumption (including compressor and pump), (c) inlet liquid flow rate, and (d) inlet gas–liquid flow rate. Reynolds number = 60.

### Gas Permeation: Process and Membrane Parameters

Figure 7a shows that the permeation area increased for all the membranes evaluated when the stage-cut was increased, which is expected. At a fixed stage-cut, the permeation area increased with selectivity. Because the permeability of CO_2_ was fixed at two values of 100 or 1000 GPU, decreasing the selectivity implies increasing the permeance of the other gases in the mixture, ultimately rendering the membrane more permeable to the gas in general.

**Figure 7:**
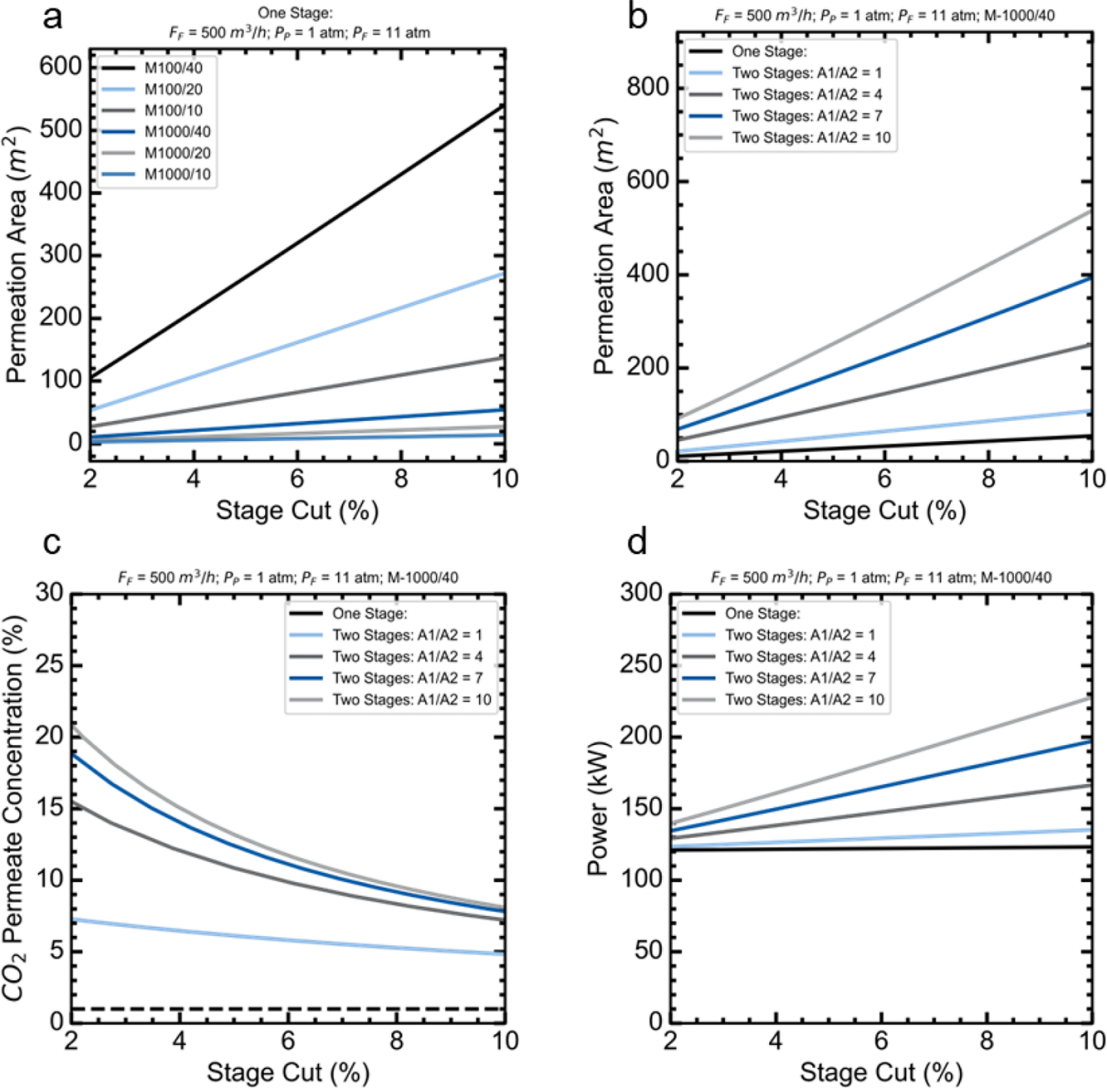
Impact of stage-cut feed, membrane characteristics, and flowsheet design on (a) the membrane contactor area, (b) the overall power consumption (including compressor and pump), (c) the input liquid flow rate, and (d) the inlet gas–liquid flow rate.

Fixing the membrane M-1000/40, Figure 7b shows the increase in the permeation area when changing the flowsheet. The one-stage flowsheet exhibited the smallest permeation area. For the two-stage flowsheet, the total permeation area increased with the ratio A_1_/A_2_. This parameter is the area ratio of the first and second gas-permeation modules in the two-stage flowsheet.

It is important to evaluate the CO_2_ concentration in the permeate stream because it will be used as the inlet stream at the membrane contactor. Depending on the flowsheet used, Figure 7c shows how the concentration of CO_2_ in the permeate stream decreased as the stage cut increased. The CO_2_ concentration in the permeate stream for the two-stage flowsheet increased when the ratio A_1_/A_2_ increased. However, the two-stage A_1_/A_2_ = 1 flowsheet presented the same CO_2_ concentration at the permeate as the one-stage flowsheet.

Figure 7d demonstrates that the power behaves similarly to the permeation area, depending on the flowsheet and stage cut.

### Process Coupling

It is important to consider the coupling process and analyze its variables to minimize the total membrane area and power consumption. This approach can significantly improve the overall operational efficiency of the system.

The direct method (Figure 7a) was used to simulate the process coupling and evaluate it at a fixed feed flow rate. Figure 8a shows that as the inlet stream pressure increased, the total membrane area (gas permeation area plus membrane contactor area) decreased. For the set of membranes with a CO_2_ permeance of 1000 GPU, increasing the membrane selectivity reduced the total membrane area. For the membrane with a CO_2_ permeance of 100 GPU, the entire membrane area increased with membrane selectivity.

**Figure 8:**
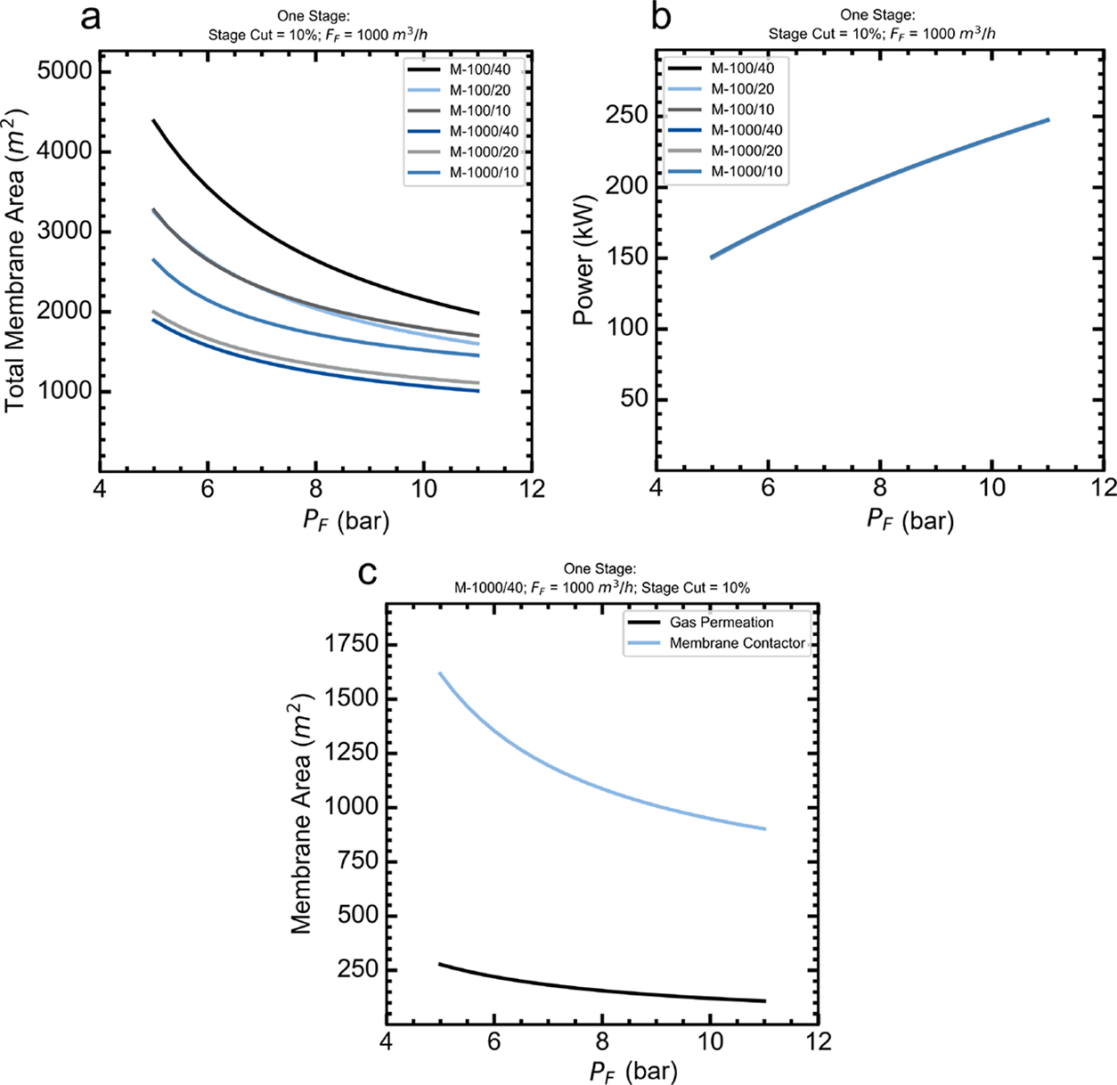
Process coupling simulations using the direct methodology. The impact of stage-cut, feed pressure, and membrane properties on (a) the overall membrane area, (b) the total power consumption (including the compressor and pump), (c) gas permeation area and membrane contactor area. Reynolds number = 60.

The M-1000/40 membrane was the best gas permeation membrane for the coupling process, achieving the lowest total membrane area ranging from 1900 to 1000 m^2^ when the feed pressure was changed from 5 to 11 bar. The worst membrane was M-100/40 because it is less permeable than the others, and achieving the same stage cut requires a larger permeation membrane area.

Figure 8b shows the total power consumed was practically the same regardless of the membrane used. The inlet gas flow rate was fixed at 1000 m^3^/h, which means that the compressor at the inlet stream from the gas permeation flowsheet consumed most of the power required for the processor operation. Selecting the membrane M-1000/40, Figure 8c shows that the permeation area is lower than the membrane contactor area, which implies that the membrane contactor has a greater influence on the total membrane area.

The inverse methodology (Figure 5b) was used to evaluate the coupling process at a fixed gas velocity in the membrane contactor. For this analysis, only the M1000/40, M100/40, and M100/20 membranes were chosen, and all gas permeation flowsheets were considered. Process coupling was compared to the membrane contactor process alone, that is, without coupling.

Figure 9 shows how the gas velocity per fiber inside the membrane contactor affects the total membrane area. For the gas velocity of 0.31 m/s, the membrane area varied from 850 to 6620 m^2^. For the gas velocity of 0.03 m/s, the membrane area varies between 3200 and 24000 m^2^.

**Figure 9:**
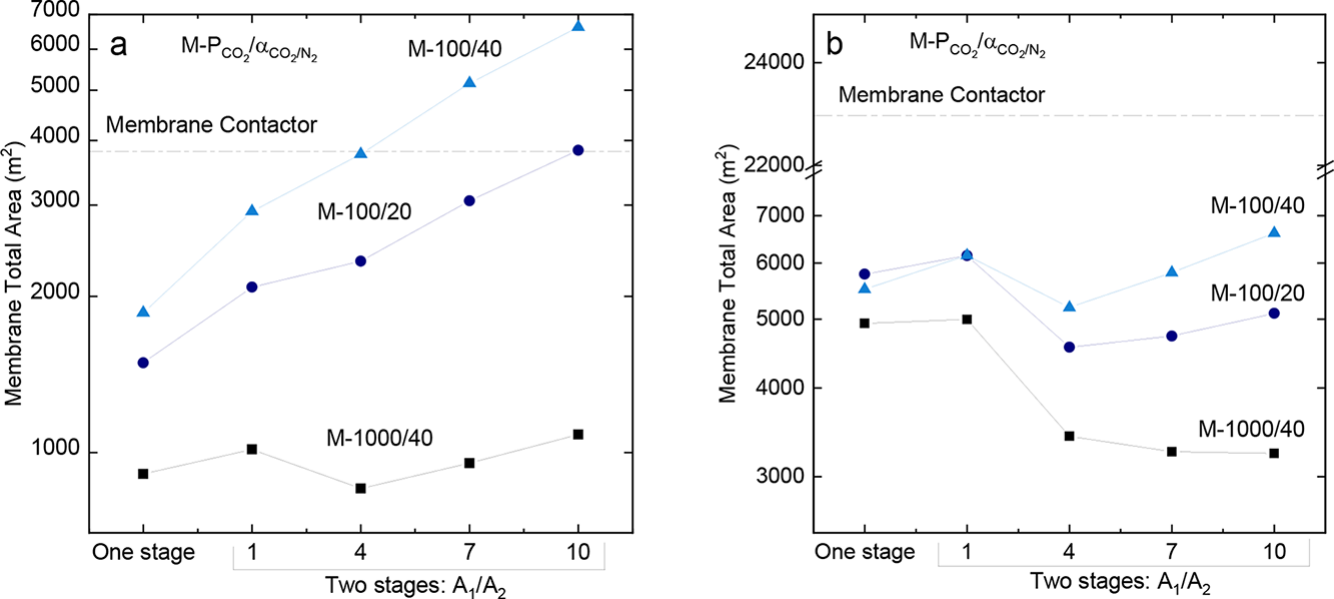
Process coupling simulations using the inverse methodology. Impact of the membrane properties and gas permeation flowsheet on the total membrane area for fixed gas velocity per fiber (a) 0.31 m/s and (b) 0.03 m/s.

Figure 9 shows that the coupled process reduces the overall membrane area compared with the membrane contactor operating independently across all scenarios at a gas velocity of 0.03 m/s. In this scenario, the low gas velocity reduces the driving inside the membrane contactor, increasing the membrane area to achieve a fixed CO_2_ removal rate. In contrast, process integration mitigates the impact of the gas velocity by enhancing the driving force of the process by increasing the CO_2_ concentration at the inlet stream of the membrane contactor module.

In contrast, the total membrane area of the process integration was smaller than that of the standalone membrane contactor when the gas velocity per fiber was 0.31 m/s. However, exceptions were observed when the M100/40 membrane was utilized in the two-stage flowsheet with A_1_/A_2_ equal to 4, 7, and 10, as well as when the M100/20 membrane was employed in the two-stage flowsheet with A_1_/A_2_ = 10. In these scenarios, a high gas velocity ensured a high driving force throughout the length of the membrane contactor module. Therefore, the increase in total membrane area was attributed to the low permeability of the M100/40 and M100/20 membranes, requiring a larger membrane surface area to achieve the desired stage cut for the gas permeation process.

The M1000/40 membrane ensured that the smallest total membrane area was obtained under all the examined conditions. For the gas velocity of 0.03 m/s, the total membrane area decreases as the ratio between the areas of the two-stage flowsheets increases (1 > 4 > 7 > 10). This behavior occurs because of the increased concentration of CO_2_, which feeds the membrane contactor from the two-stage flowsheet. Using the one-stage flowsheet, it is possible to achieve a slightly smaller area than the A_1_/A_2_ = 1 two-stages flowsheet but a larger area than the other two-stage arrangements.

For a gas velocity of 0.31 m/s, the smallest area obtained was 852 m^2^ and occurred when the M1000/40 membrane was applied in a A_1_/A_2_ = 4. It is important to note that the one-stage arrangement had a membrane area of 909 m^2^, which is only 6.7% higher than the minimum value. The one-stage flowsheet is simpler to build and operate than the two-stage flowsheet. In this sense, using two stages would not be worthwhile because the space economy gain would be minimal compared to the increased complexity of the process assembly and operation.

## Operational Curve

To evaluate the impact of the gas permeation membrane properties (permeance and selectivity) on the total membrane area of the integrated process, the inverse approach was applied, and all the process variables were fixed: the feed pressure was set at 10 bar, the stage cut was set at 10%, and the Reynolds number was set to 60. The gas permeation flowsheet considers only one stage, as it presents lower areas and is simpler than the others. The gas velocity was fixed at 0.03 and 0.31 m/s.

This analysis aimed to examine the transport parameters of the gas permeation membrane to obtain a system operating curve based on the membrane permeability and selectivity. This is important because it can guide commercial membrane selection and/or new membrane development.

Figure 10 shows the results of the analysis using contour graphics containing comertial membrane and literature data.^31,32^ By analyzing the results, it is possible to note the significant variation in the total membrane area, which ranges from 660 to 9200 m^2^. The lowest area achieved when applying the lower gas velocity was 4400 m^2^. For the highest gas velocity, the minimal membrane area corresponds to 660 m^2^.

**Figure 10:**
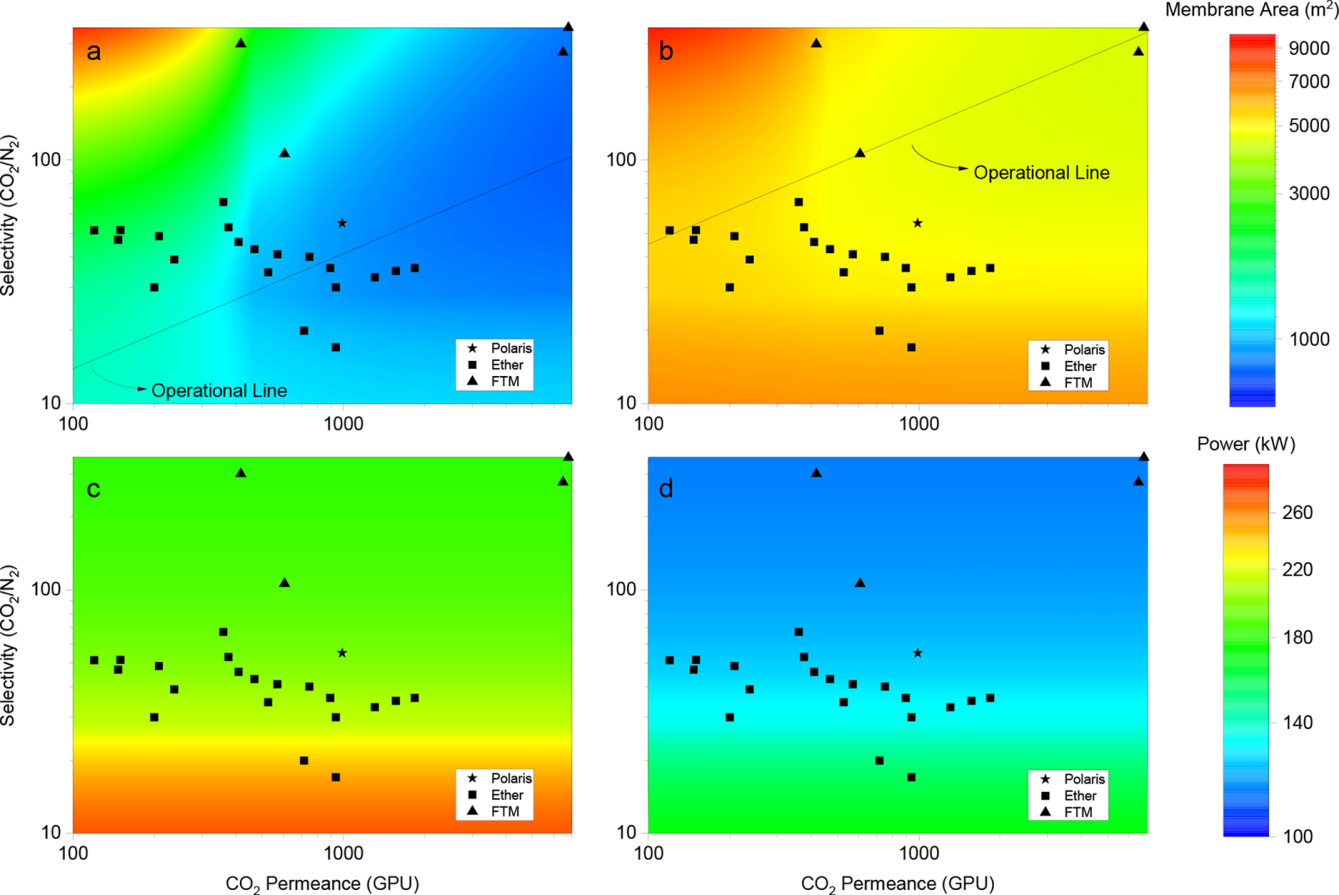
Process coupling simulation using the inverse methodology. Influence of gas permeation membrane properties on the total membrane area (a, b) and total power consumption (c, d) for two different gas velocities per fiber inside the membrane contactor: (a, c) 0.31 m/s and (b, d) 0.03 m/s. Solid lines represent the operational curve, while scattered points correspond to commercial membranes and selected literature data. Adapted with permission from Han and Ho (2021). Polymeric membranes for CO_2_ separation and capture. Journal of Membrane Science, 628, 119244. Copyright 2021 Elsevier.

The operational working curve considers the lowest total membrane area achieved for the specific permeance and selectivity pairs of membranes. Figure 10b shows the operational curve (solid black line) within a selectivity range of 45–350. Only the facilitated transport membranes (FTM) can operate in this condition. None of the other membranes, including the commercial Polaris membrane, performed optimally. Highly selective membranes are necessary to offset the decrease in the driving force inside the membrane contactor caused by the slow gas velocity and increased feed concentration at this stage.

However, considering the highest gas velocity, Figure 10a shows that the operating curve (solid black line) is limited to a selectivity range of 10–100. The FTM failed to perform effectively under this high-speed gas situation. The polyester membranes and commercial Polaris membrane exhibit transport properties that closely align with the operational curve of the process in a particular situation. The high gas velocity in the membrane contactors guarantees minimal variation in the driving force along its length during the gas–liquid absorption process. When the membrane selectivity increased beyond the working curve, the overall permeability of the membrane decreased. Consequently, it is necessary to increase the gas permeation area. On the contrary, when the selectivity decreases below the working curve, the permeate stream supplied to the membrane contactors becomes less concentrated in CO_2_, increasing the membrane area during the liquid gas absorption stage.

The total power consumed by the combined process decreases with increasing membrane selectivity regardless of CO_2_ permeance (Figure 10c,d). The composition of the permeate stream produced by the gas permeation model depends only on the selectivity of the membrane, which means that the permeance will only affect the permeation area when a fixed state cut is used. Because the membrane contactor receives the permeate stream as an inlet stream, and the gas velocity per fiber is fixed, the membrane contactor area is the same for a fixed selectivity value of the gas permeation membrane. This implies the same total gas flow rate at gas permeation and the same power consumed because of the predominance of the compressor at the inlet stream of the gas permeation.

## Conclusions

This study explored the integration of gas permeation and membrane contactors as an effective approach for CO_2_ removal in confined environments. Computational modeling demonstrated that optimizing membrane properties, particularly permeance and selectivity, significantly enhanced process performance while reducing the system footprint. The results indicate that membranes with high CO_2_ permeance and selectivity contribute to process intensification by lowering the energy consumption and minimizing the required membrane area. Furthermore, the study highlights the importance of proper process design, showing that a single-stage flowsheet can achieve performance comparable to that of multistage systems while simplifying operations. These findings provide valuable insights into membrane development, guiding future research toward materials with improved transport properties for enhanced CO_2_ separation. Future work should focus on the experimental validation of the proposed hybrid process and on the fabrication of advanced membranes with tailored selectivity and permeability to optimize performance. This study contributes to the advancement of CO_2_ capture technologies for controlled atmosphere applications.

## Supplementary Material



## Footnotes

1 One GPU = 10^–6^ cm^3^(STP)/(cm^2^ s cmHg) = 2.7 × 10^–3^ m^3^(STP)/(m^2^ h bar).

## Notes

Additional experimental details, equations, and validation data for the gas permeation and membrane contactor models, including tables and figures (PDF)
