# Supplementary material for: Process Intensification by Coupling Gas Permeation and Membrane Contactor for Removing CO2 at Low Partial Pressure
Source: ACS Omega. 2025 Dec 15;10(51):62441–51. doi: 10.1021/acsomega.4c11526 (PMC12756804; doi:10.1021/acsomega.4c11526)
Supplement: Supplementary file 1 [file ao4c11526_si_001.pdf]

# Supporting Information: Process Intensification by Coupling Gas Permeation and Membrane Contactor for Removing $CO_2$ at Low Partial Pressure

Felipe B. de S. Mendes,*,†,‡ Cristina C. Pereira,† Paulo C. Sedrez,† Priscila Simões T. Amaral,† and Cristiano P. Borges‡

†*Brazilian Navy Research Institute, Rio de Janeiro, RJ, Brazil*

‡*COPPE/Chemical Engineering Program, Federal University of Rio de Janeiro, Brazil*

E-mail: felipe.mendes@marinha.mil.br

# Equations

$$A_{contactor} = \pi.n_{fiber}.d_{fiber}.L_{fiber} \tag{S1a}$$

$$v = \frac{R^2}{16.\mu_{gas}.L_{fiber}}.\frac{P_I^2 - P_{II}^2}{P_I} \tag{S1b}$$

$$KG\left(i\right) = \frac{Sh\left(i\right).D\left(i\right)}{d_h} \tag{S1c}$$

$$Sh\left(i\right) = 1.25.\left(\frac{Re.d_h}{L_{fiber}}\right)^{0.93}\left(Sc(i)\right)^{\frac{1}{3}} \tag{S1d}$$

$$Sc(i) = \frac{\mu_{Liq}(T_{Liq}, S_{Liq})}{\rho_{Liq}(T_{Liq}, S_{Liq}, P_{Liq}).D(i)} \tag{S1e}$$

$$Re = 4.\left(\frac{Q_{Liq}}{P_m}\right).\left(\frac{\rho_{Liq}(T_{Liq}, S_{Liq}, P_{Liq})}{\mu_{Liq}(T_{Liq}, S_{Liq})}\right) \tag{S1f}$$

$$\tag{S1g}$$

# Membrane contactor

## Module

Table S1: Minntech commercial module specification (data provided by manufacturer)

| | Parameter | FiberFlo |
|---|---|---|
| Fiber | Outer diameter, OD ($\mu$m) | 300 |
| | Inner diameter, ID ($\mu$m) | 240 |
| | Material | PP |
| | Mean porous diameter, PD (nm) | 30 |
| | Porosity, $\epsilon$ (%) | 30 |
| | Tortuosity, $\tau$ | 2,6 |
| Module | Length, L (cm) | 10 |
| | Number of fibers, n | $\approx$ 2200 |
| | Area, A ($m^2$) | 0,2 |
| | Packing density ($m^2/m^3$) | 3947 |
| | Shell diameter, SD (cm) | 2,54 |

# Experimental Methodology

This methodology was employed by Mendes[1] to evaluate the $CO_2$ absorption using membrane contactor and salty alkaline water as liquid absorbent.

The membrane contactor were evaluated in the experimental apparatus depicted at Figure S1, exploring the conditions listed in Table S2. The unit has a membrane module (M-1) and allows the control of the gas mixture composition and flow rate by a mass flow controller. The pH of the liquid phase was controlled by NaOH addition, and its temperature was kept constant by a thermostatic bath. The gas composition was monitored online by gas chromatography.

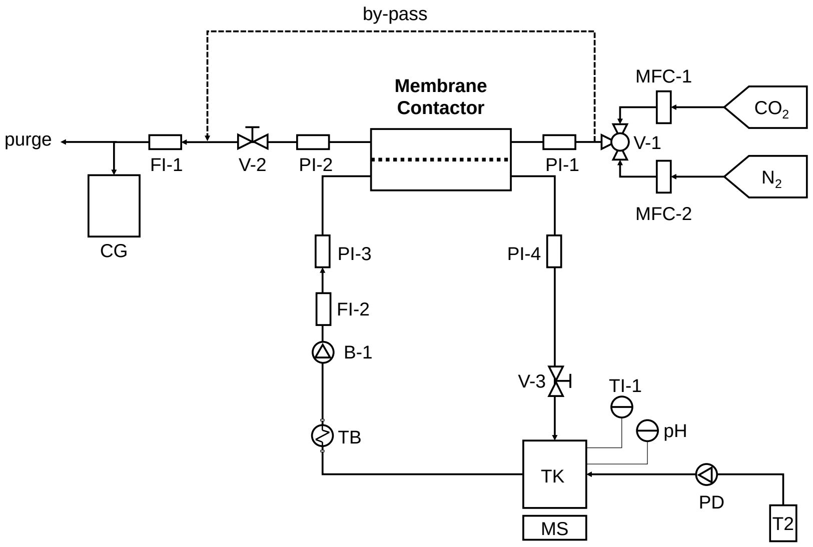


Figure S1: Flowsheet of the MC performance test. CG – chromatograph, FI – flow indicator, V-valves; PI – pressure indicator; B-01 – liquid pump; TB – thermostatic bath; TK – liquid tank; MS – magnetic stirrer; TI – temperature indicator; pH – pH indicator; PD – dosing pump; (T-2 : NaOH solution); MFC – mass flow controller.

The flux was calculated by Equation S2 and selectivity was the ratio of the fluxes.

$$J_k = \frac{P_{\text{gas}}}{R \cdot T_{\text{gas}}} \left( \frac{Q_{\text{gas}_{k,i}} \cdot Y_{k,i} - Q_{\text{gas}_{k,o}} \cdot Y_{k,o}}{A} \right) \tag{S2}$$

where $J_k$ is the molar flux of component k [mol/m2.s], $P_{gas}$ is absolute pressure of the gas stream [bar], $T_{\text{gas}}$ is temperature of the gas stream [K], $R$ is universal gas constant [$cm^3$. bar/

mol. K], $Q_{\text{gas}_k}$ is volumetric flow rate of component k [$cm^3$/min] and $Y_k$ is the volumetric fraction of component k in the gas stream. The subscripts i and o means input and output from the module.

A stream containing 10% of $CO_2$ in nitrogen at a flow rate of 100 $cm^3$/min (2 bar absolute and 298 K) was used in all experiments conditions reported in Table 2. The absorbent was designed to be as close as possible to seawater conditions, so that pH was kept at 8 during all experiments and NaCl content at 3.5%wt. All experiments were carried out in counter-current where the gas stream flows inside and the liquid stream flows outside the fibers.

Table S2: Membrane contactor experimental conditions

| # | Gas Pressure (bar*) | Liquid Pressure (bar*) | NaCl (%wt) | Flow rate (L/h) | Temperature (ºC) |
|---|---|---|---|---|---|
| 1 | 2 | 2.2 | 3.5 | 20 | 10 |
| 2 | 2 | 2.2 | 3.5 | 20 | 20 |
| 3 | 2 | 2.2 | 3.5 | 20 | 30 |
| 4 | 2 | 2.2 | 3.5 | 50 | 10 |
| 5 | 2 | 2.2 | 3.5 | 50 | 20 |
| 6 | 2 | 2.2 | 3.5 | 50 | 30 |

# Gas Permeation Membranes

Table S3: Transport properties of gas permeation membranes, including selected data from Merkel et al.[2], Han and Ho[3].

| **Material** | **p($CO_2$) [atm]** | **T [°C]** | **P($CO_2$) [Barrer]** | $\alpha$ **[$CO_2/N_2$]** |
|---|---|---|---|---|
| PEO-b-PA6 | 10 | 35 | 120 | 51.4 |
| PEO-b-PBT | 0.3 | 30 | 150 | 51.5 |
| POEM-g-PVC | 1 | 35 | 147 | 47 |
| PEO-ran-PPO-b-T6T6T | 4 | 35 | 470 | 43 |
| PEO-PBT/PEG200 | 0.3 | 30 | 208 | 48.7 |
| PEO-PBT/PEG-DBE | 0.3 | 30 | 750 | 40 |
| Pebax®1074/PEG1500 | 5 | 60 | 528 | 34.6 |
| PEO-PPO-T6T6T/PDMS-PEG | 4 | 35 | 896 | 36 |
| Pebax®/PGP-POEM | 1 | 35 | 237 | 39 |
| Pebax®1657/PEGDME500 | 0.17 | 57 | 940* | 30 |
| Pebax® 2533/PEG-b-PPFPA | 3.5 | 35 | 940* | 17 |
| PEGDA/PEGMEA | 4 | 35 | 570 | 41 |
| PEGDA/TRIS-A | 15 | 35 | 716 | 19.9 |
| TEGMVE/VEEM | 1 | 25 | 410 | 46 |
| PEO-526/dopamine/PEGDME | 3.5 | 50 | 200 | 30 |
| PEO-amine/PEO-epoxy | 3 | 35 | 376 | 53 |
| GPA1000-g-PEG-azide | 2 | 45 | 1840 | 36 |
| PEO-POSS-NH2 | 3.5 | 35 | 1567 | 35 |
| PEA/TMC | 0.2 | 25 | 360* | 67.2 |
| DGBAmE/TMC | 0.71 | 22 | 1310* | 33 |
| PVAm-CH3 | 0.04 | 102 | 6804 | 350 |
| PEI/PVA | 0.04 | 25 | 418 | 300 |
| PVAm/EDA | 0.02 | 30 | 607* | 106 |
| PVAm/PZ | 0.02 | 22 | 6503* | 277 |
| Polaris® | - | - | 1000* | 50 |

* permeance in GPU measured for a thin-film composite membrane (TFC)

# Model Validation Data

## Membrane Contactor

Table S4: The parameters of the equation describing the Enrichment Factor are estimated as follows: A = 0.039. B = -1044.58. and the Objective Function is equal to 0.044. Data obtained from Mendes[1].

| Experiment | Experimental Flux ($mol.m^{-2}.s^{-1}$) | Calculated Flux ($mol.m^{-2}.s^{-1}$) | Error (%) |
|---|---|---|---|
| 1 | 1.96 x $x10^{-5}$ | 1.83 x $x10^{-5}$ | -6.63 |
| 2 | 1.63 x $x10^{-5}$ | 1.64 x $x10^{-5}$ | 0.61 |
| 3 | 1.49 x $x10^{-5}$ | 1.50 x $x10^{-5}$ | 0.67 |
| 4 | 3.47 x $x10^{-5}$ | 3.58 x $x10^{-5}$ | 3.17 |
| 5 | 3.35 x $x10^{-5}$ | 3.29 x $x10^{-5}$ | -1.79 |
| 6 | 3.04 x $x10^{-5}$ | 3.06 x $x10^{-5}$ | 0.66 |

## Gas Permeation

Table S5: Parameters used for validating the gas permeation model. Data compiled from Brinkmann et al.[4],[5].

| $\mathbf{Q_F}$ [Nm3/h] | $\mathbf{P_F}$ [bar] | $\mathbf{P_P}$ [bar] | $\mathbf{z_P(CO_2)}$ | Perm($CO_2$ )* [GPU] | Perm($N_2$)* [GPU] | A [$m^2$] | Ref. |
|---|---|---|---|---|---|---|---|
| 26.31 | 4.32 | 0.1 | 0.182 | 1142.00 | 19.92 | 0 a 10 | [4] |
| 34.24 | 2.55 | 0.2 | 0.174 | 1142.00 | 19.92 | 0 a 10 | [4] |
| 31.93 | 3.03 | 0.102 | 0.173 | 1965.89 | 37.42 | 6.26 | [5] |
| 38.75 | 3.02 | 0.104 | 0.173 | 1965.89 | 37.42 | 6.26 | [5] |
| 47.25 | 3.08 | 0.11 | 0.169 | 1965.89 | 37.42 | 6.26 | [5] |

* Permeances were calculated based on data from Brinkmann et al.[4],[5].

Table S6: Comparison of the model proposed in this study with mathematical models presented by Brinkmann et al.[4].

| Model | $\mathbf{z_P(CO_2)}$ | ***stage-cut*[%]** | Deviation $\mathbf{z_P(CO_2)}$ | Deviation Stage-Cut |
|---|---|---|---|---|
| Envelope | 0.7668 | 14.16 | -6.64% | -7.95% |
| Spiral wound | 0.7589 | 13.80 | -5.67% | -5.55% |
| Countercurrent | 0.7536 | 13.85 | -5.01% | -5.89% |
| Countercurrent 4 segments | 0.7627 | 14.32 | -6.14% | -8.98% |
| This work | 0.7159 | 13.03 | - | - |

# References


(1) Mendes, F. Remoção de CO2 de Ambientes Confinados Utilizando Contactores com Membranas e agua do Mar Sintética como Absorvente. 2017; `https://pantheon.ufrj.br/bitstream/11422/10288/1/877215.pdf`.

(2) Merkel, T. C.; Lin, H.; Wei, X.; Baker, R. Power plant post-combustion carbon dioxide capture: An opportunity for membranes. *Journal of Membrane Science* **2010**, *359*, 126–139, Membranes and CO2 Separation.

(3) Han, Y.; Ho, W. W. Polymeric membranes for CO2 separation and capture. *Journal of Membrane Science* **2021**, *628*, 119244.

(4) Brinkmann, T.; Pohlmann, J.; Withalm, U.; Wind, J.; Wolff, T. Theoretical and Experimental Investigations of Flat Sheet Membrane Module Types for High Capacity Gas Separation Applications. *Chemie Ingenieur Technik* **2013**, *85*, 1210–1220.

(5) Brinkmann, T.; Notzke, H.; Wolff, T.; Zhao, L.; Luhr, S.; Stolten, D. Characterization of a New Flat Sheet Membrane Module Type for Gas Permeation. *Chemie Ingenieur Technik* **2019**, *91*, 30–37.
